# Hospitalizations of Children Aged 5–11 Years with Laboratory-Confirmed COVID-19 — COVID-NET, 14 States, March 2020–February 2022

**DOI:** 10.15585/mmwr.mm7116e1

**Published:** 2022-04-22

**Authors:** Dallas S. Shi, Michael Whitaker, Kristin J. Marks, Onika Anglin, Jennifer Milucky, Kadam Patel, Huong Pham, Shua J. Chai, Breanna Kawasaki, James Meek, Evan J. Anderson, Andy Weigel, Justin Henderson, Ruth Lynfield, Susan L. Ropp, Alison Muse, Sophrena Bushey, Laurie M. Billing, Melissa Sutton, H. Keipp Talbot, Andrea Price, Christopher A. Taylor, Fiona P. Havers, COVID-NET Surveillance Team

**Affiliations:** ^1^CDC COVID-19 Emergency Response Team; ^2^Epidemic Intelligence Service, CDC; ^3^General Dynamics Information Technology, Atlanta, Georgia; ^4^California Emerging Infections Program, Oakland, California; ^5^Career Epidemiology Field Officer Program, CDC; ^6^Colorado Department of Public Health and Environment; ^7^Connecticut Emerging Infections Program, Yale School of Public Health, New Haven, Connecticut; ^8^Emory University School of Medicine, Atlanta, Georgia; ^9^Georgia Emerging Infections Program, Georgia Department of Health; ^10^Atlanta Veterans Affairs Medical Center, Atlanta, Georgia; ^11^Iowa Department of Public Health; ^12^Michigan Department of Health and Human Services; ^13^Minnesota Department of Health; ^14^New Mexico Department of Health; ^15^New York State Department of Health; ^16^University of Rochester School of Medicine and Dentistry, Rochester, New York; ^17^Ohio Department of Health; ^18^Public Health Division, Oregon Health Authority; ^19^Vanderbilt University Medical Center, Nashville, Tennessee; ^20^Salt Lake County Health Department, Salt Lake City, Utah.

On October 29, 2021, the Food and Drug Administration expanded the Emergency Use Authorization for Pfizer-BioNTech COVID-19 vaccine to children aged 5–11 years; CDC’s Advisory Committee on Immunization Practices’ recommendation followed on November 2, 2021.* In late December 2021, the B.1.1.529 (Omicron) variant of SARS-CoV-2 (the virus that causes COVID-19) became the predominant strain in the United States,^†^ coinciding with a rapid increase in COVID-19–associated hospitalizations among all age groups, including children aged 5–11 years (*1*). COVID-19–Associated Hospitalization Surveillance Network (COVID-NET)^§^ data were analyzed to describe characteristics of COVID-19–associated hospitalizations among 1,475 U.S. children aged 5–11 years throughout the pandemic, focusing on the period of early Omicron predominance (December 19, 2021–February 28, 2022). Among 397 children hospitalized during the Omicron-predominant period, 87% were unvaccinated, 30% had no underlying medical conditions, and 19% were admitted to an intensive care unit (ICU). The cumulative hospitalization rate during the Omicron-predominant period was 2.1 times as high among unvaccinated children (19.1 per 100,000 population) as among vaccinated^¶^ children (9.2).** Non-Hispanic Black (Black) children accounted for the largest proportion of unvaccinated children (34%) and represented approximately one third of COVID-19–associated hospitalizations in this age group. Children with diabetes and obesity were more likely to experience severe COVID-19. The potential for serious illness among children aged 5–11 years, including those with no underlying health conditions, highlights the importance of vaccination among this age group. Increasing vaccination coverage among children, particularly among racial and ethnic minority groups disproportionately affected by COVID-19, is critical to preventing COVID-19-associated hospitalization and severe outcomes.

COVID-NET conducts population-based surveillance for laboratory-confirmed COVID-19–associated hospitalizations in 99 counties across 14 U.S. states.^††^ COVID-19–associated hospitalizations are defined as receipt of a positive SARS-CoV-2 nucleic acid amplification tests or rapid antigen detection test result during hospitalization or during the 14 days preceding admission. This analysis describes hospitalization rates among children aged 5–11 years during March 1, 2020–February 28, 2022. Clinical data from the Omicron-predominant period were compared with those from the Delta-predominant (June 27–December 18, 2021) and pre-Delta (March 1, 2020–June 26, 2021) periods; a variant that accounted for >50% of sequenced isolates was considered predominant. Unadjusted weekly COVID-19–associated hospitalization rates (COVID-19–related hospitalizations per 100,000 children) were calculated by dividing the total number of COVID-19–associated hospitalizations by the population estimates for the counties included in the surveillance area.^§§^ ICU admission rates were calculated using 2-week periods. Population-based hospitalization rates and data for hospitalized children were compared by COVID-19 vaccination status for the Omicron-predominant period using linkage to state immunization information systems data.^¶¶^

Trained surveillance officers abstracted medical charts for hospitalized pediatric patients using standardized case report forms through November 2021. Because of the surge in hospitalizations during December 2021–February 2022, some sites examined clinical data on a representative sample of hospitalized children during this period.*** The representative sample included 1,252 of 1,475 (84.9%) children with positive SARS-CoV-2 test results; complete clinical data were available for 595 of 596 (99.8%), 438 of 468 (93.6%), and 219 of 225 (97.3%) sampled children aged 5–11 years during the pre-Delta period, Delta-predominant period, and Omicron-predominant period.

Data regarding likely primary reason for hospital admission,^†††^ symptoms at admission,^§§§^ underlying medical conditions,^ ¶¶¶ ^ vaccination status (complete versus incomplete), and indicators of severe disease (e.g., length of stay, ICU admission, receipt of invasive mechanical ventilation [IMV],**** and in-hospital death) were collected (*2*). Children who completed their primary COVID-19 vaccination series were defined as those who had received the second dose of a 2-dose series ≥14 days before receipt of a positive SARS-CoV-2 test result associated with their hospitalization. Wilcoxon rank-sum tests and chi-square tests were used to compare medians and proportions, respectively; p<0.05 was considered statistically significant. Percentages were weighted to account for probability of selection for sampled cases and adjusted to account for nonresponse. Association of underlying medical conditions with severe COVID-19 (defined as requiring ICU admission or IMV, or in-hospital death) was modeled using multivariable generalized estimating equations (*2*). Multivariable models were limited to children whose primary reason for admission was likely COVID-19–related. Unadjusted risk ratios (RRs), adjusted RRs (aRRs), and 95% CIs were calculated for the association of demographic characteristics, underlying medical conditions, and variant periods with severe COVID-19. Data were analyzed using SAS (version 9.4; SAS Institute). This activity was reviewed by CDC and conducted consistent with applicable federal law and CDC policy.^ ††††^

During the Delta- and Omicron-predominant periods, weekly hospitalization rates of children aged 5–11 years peaked during the weeks ending September 25, 2021 and January 22, 2022, respectively; the Omicron-predominant peak (2.8 per 100,000 children) was 2.3 times the Delta-predominant peak (1.2).^§§§§^ Peak ICU admission rates were 1.7 times as high during Omicron predominance (2-week period ending January 25, 2022 [1.2]) than during Delta predominance (2-week period ending October 2, 2021 [0.7]). 

During the Omicron-predominant period, cumulative hospitalization rates among unvaccinated children aged 5–11 years were 2.1 times as high (19.1) as those among vaccinated children (9.2) (Figure). Most (87%) children aged 5–11 years hospitalized during the Omicron-predominant period were unvaccinated (Supplemental Table, https://stacks.cdc.gov/view/cdc/116353). Among unvaccinated children, the largest proportion were Black (34%), followed by White (31%), and Hispanic (19%). There were no significant differences for severe outcomes by vaccination status. However, the number of vaccinated children was small. No vaccinated children required higher level O_2_ support (e.g., bilevel positive airway pressure/continuous positive airway pressure [BiPAP/CPAP], high flow nasal canula, or IMV).

**FIGURE:**
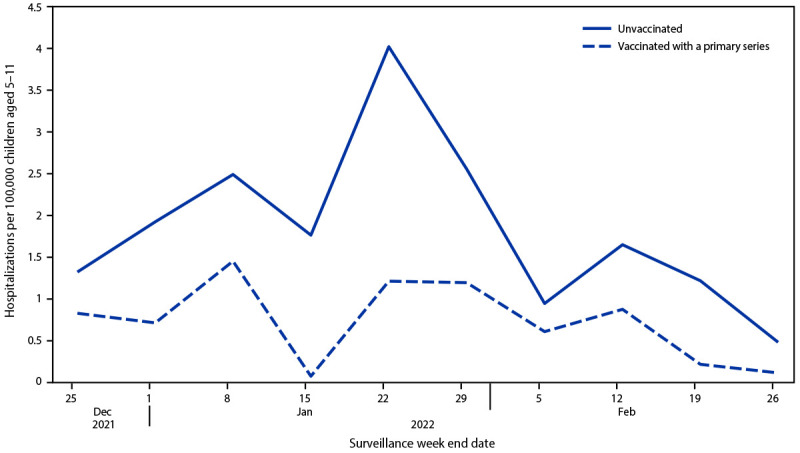
Weekly COVID-19–associated hospitalization rates* among children aged 5–11 years, by vaccination status^†^ during the Omicron-predominant period — COVID-NET,^§^ 11 states, December 25, 2021– February 26, 2022 **Abbreviation:** COVID-NET = COVID-19–Associated Hospitalization Surveillance Network. * Number of children aged 5–11 years with laboratory-confirmed COVID-19–associated hospitalizations per 100,000 population; rates are subject to change as additional data are reported. ^† ^Children who completed their primary COVID-19 vaccination series were defined as those who had received the second dose of a 2-dose series ≥14 days before receipt of a positive SARS-CoV-2 test result associated with their hospitalization. ^§^ COVID-NET sites during the period shown are in the following 11 states: California, Colorado, Connecticut, Georgia, Minnesota, New Mexico, New York, Ohio, Oregon, Tennessee, and Utah.

COVID-19–related illness was the primary reason for admission among a lower proportion of hospitalized children aged 5–11 years during the Omicron period (73%) compared with the Delta period (84%) (p<0.01); across all periods, a majority (78%) of children were hospitalized with COVID-19 as the likely primary reason for admission (Table 1). Of the hospitalized children, 67% had one or more underlying medical conditions. During the period of Omicron predominance, a larger proportion of children hospitalized with COVID-19 had neurologic disorders (33%) compared with those hospitalized during the pre-Delta period (21%) (p<0.01), and a lower proportion had obesity (33% and 21%, respectively; p = 0.01). Similar trends were observed when comparing the Omicron- and Delta-predominant periods. Among children hospitalized during the Omicron-predominant period, 19% required ICU admission, including 15% with no underlying medical conditions; 5% received IMV; none died.

**TABLE 1:** Demographic and clinical characteristics and outcomes among children aged 5–11 years with laboratory-confirmed COVID-19, by variant period — COVID-NET, 14 states,* March 1, 2020–February 28, 2022

Characteristic	Variant period, no. (%) of hospitalizations^†^				p-value^§^ (Omicron versus pre-Delta)	p-value^§^ (Omicron versus Delta)
	Total	Pre-Delta Mar 1, 2020–Jun 26,2021	Delta predominant Jun 27, 2021–Dec 18, 2021	Omicron predominant Dec 19, 2021–Feb 28, 2022		
**Total no. of hospitalized children**	**1,475^¶^**	**596** ^¶^	**482** ^¶^	**397** ^¶^	**NA**	**NA**
**Age, yrs, median (IQR)**	**8 (6–10)**	8 (6–10)	9 (6–10)	8 (6–10)	0.03	0.01
**Sex**						
Male	**829 (56.2)**	353 (59.2)	258 (53.6)	218 (54.9)	0.18	0.71
Female	**645 (43.8)**	243 (40.8)	223 (46.4)	179 (45.1)		
Race/Ethnicity**						
White, non-Hispanic	**430 (29.2)**	129 (21.6)	163 (33.9)	138 (34.8)	<0.01	0.42
Black, non-Hispanic	**484 (32.8)**	197 (33.1)	167 (34.7)	120 (30.2)		
Asian or Pacific Islander, non-Hispanic	**64 (4.3)**	24 (4.0)	19 (4.0)	21 (5.3)		
Hispanic	**420 (28.5)**	212 (35.6)	114 (23.7)	94 (23.7)		
Persons of all other races^††^	**26 (1.8)**	14 (2.3)	6 (1.2)	6 (1.5)		
Unknown race/ethnicity	**50 (3.4)**	20 (3.4)	12 (2.5)	18 (4.5)		
**Primary reason for admission^§§^**						
Likely COVID-19–related	**944 (78.2)**	420 (76.7)	364 (84.2)	160 (72.9)	0.31	<0.01
**Underlying medical conditions**						
One or more underlying medical condition^¶¶^	**824 (66.7)**	383 (64.9)	288 (66.6)	153 (69.6)	0.25	0.48
Obesity	**302 (29.0)**	152 (33.0)	111 (30.6)	39 (21.3)	0.01	0.03
Neurologic disorder***	**306 (25.3)**	124 (21.0)	106 (24.5)	76 (33.4)	<0.01	0.02
Asthma	**282 (22.4)**	133 (22.6)	100 (23.1)	49 (21.4)	0.73	0.63
Chronic lung disease, not including asthma^†††^	**130 (10.5)**	62 (10.6)	41 (9.5)	27 (11.4)	0.74	0.46
Cardiovascular disease^§§§^	**141 (11.8)**	53 (9.1)	55 (13.0)	33 (14.9)	0.02	0.50
Blood disorder^¶¶¶^	**111 (9.1)**	47 (8.0)	42 (9.9)	22 (9.9)	0.43	0.99
Immunocompromising conditions****	**117 (10.0)**	49 (8.4)	38 (9.1)	30 (13.8)	0.03	0.09
Feeding tube dependence	**78 (6.5)**	32 (5.4)	25 (6.0)	21 (9.0)	0.07	0.18
Diabetes mellitus	**58 (5.0)**	24 (4.1)	18 (4.1)	16 (7.7)	0.06	0.07
Chronic metabolic disease, not including diabetes mellitus^††††^	**40 (3.3)**	11 (1.9)	19 (4.6)	10 (3.9)	0.09	0.69
Rheumatologic/Autoimmune/Inflammatory disorders^§§§§^	**44 (3.6)**	19 (3.2)	16 (3.7)	9 (4.2)	0.54	0.79
GI/Liver disease^¶¶¶¶^	**35 (2.9)**	17 (3.0)	15 (3.5)	3 (2.1)	0.59	0.42
Renal disease*****	**29 (2.4)**	11 (1.8)	11 (2.7)	7 (3.2)	0.25	0.77
Genetic disease^†††††^	**27 (2.2)**	11 (1.9)	7 (1.6)	9 (3.7)	0.13	0.09
**Viral codetections^§§§§§^**						
Positive test results	**85 (12.3)**	33 (12.3)	37 (14.6)	15 (9.7)	0.43	0.17
**Hospitalization outcomes^¶¶¶¶¶^**						
Length of hospital stay, days, median (IQR)	**3 (2–5)**	3 (2–6)	3 (1–5)	3 (1–5)	0.01	0.54
ICU admission	**349 (27.0)**	191 (32.6)	114 (26.1)	44 (18.9)	<0.01	0.05
Invasive mechanical ventilation	**79 (6.2)**	40 (6.7)	29 (6.8)	10 (4.6)	0.28	0.28
In-hospital death	**4 (0.3)**	4 (0.7)	0 (—)	0 (—)	—	—

**Abbreviations:** COVID-NET = COVID-19–Associated Hospitalization Surveillance Network; GI = gastrointestinal; ICU = intensive care unit; NA = not applicable.

* Includes persons admitted to a hospital during March 1, 2020–February 28, 2022. Maryland contributed data through November 26, 2021. Counties included in COVID-NET surveillance during this period: California (Alameda, Contra Costa, and San Francisco counties); Colorado (Adams, Arapahoe, Denver, Douglas, and Jefferson counties); Connecticut (Middlesex and New Haven counties); Georgia (Clayton, Cobb, DeKalb, Douglas, Fulton, Gwinnett, Newton, and Rockdale counties); Iowa (one county represented); Maryland (Allegany, Anne Arundel, Baltimore, Baltimore City, Calvert, Caroline, Carroll, Cecil, Charles, Dorchester, Frederick, Garrett, Harford, Howard, Kent, Montgomery, Prince George’s, Queen Anne’s, St. Mary’s, Somerset, Talbot, Washington, Wicomico, and Worcester counties); Michigan (Clinton, Eaton, Genesee, Ingham, and Washtenaw counties); Minnesota (Anoka, Carver, Dakota, Hennepin, Ramsey, Scott, and Washington counties); New Mexico (Bernalillo, Chaves, Doña Ana, Grant, Luna, San Juan, and Santa Fe counties); New York (Albany, Columbia, Genesee, Greene, Livingston, Monroe, Montgomery, Ontario, Orleans, Rensselaer, Saratoga, Schenectady, Schoharie, Wayne, and Yates counties); Ohio (Delaware, Fairfield, Franklin, Hocking, Licking, Madison, Morrow, Perry, Pickaway and Union counties); Oregon (Clackamas, Multnomah, and Washington counties); Tennessee (Cheatham, Davidson, Dickson, Robertson, Rutherford, Sumner, Williamson, and Wilson counties); and Utah (Salt Lake County).

^† ^Data are from a weighted sample of hospitalized children with completed medical record abstractions. Sample sizes presented are unweighted with weighted percentages.

^§^ Proportions between the Omicron and Delta- and Omicron-predominant and pre-Delta periods were compared using chi-square tests, and medians were compared using Wilcoxon rank-sum tests; p<0.05 was considered statistically significant.

^¶^ Data are missing for <3% of observations for all variables.

** If ethnicity was unknown, non-Hispanic ethnicity was assumed.

^††^ Includes non-Hispanic persons reported as other or multiple races.

^§§^ Primary reason for admission was collected beginning June 1, 2020; hospitalizations before June 1, 2020 (42) are excluded. Among sampled patients, COVID-NET collects data on the primary reason for admission to differentiate hospitalizations of patients with laboratory-confirmed SARS-CoV-2 infection who are likely admitted primarily for COVID-19 illness rather than for other reasons. During chart review, if the surveillance officer finds that the chief complaint or history of present illness mentions fever or respiratory illness, COVID-19–like illness, or suspected COVID-19, then the case is categorized with COVID-19–related illness as the primary reason for admission. Reasons for admission that are likely primarily not related to COVID-19 include the following categories: inpatient surgery or procedures, psychiatric admission requiring acute medical care, trauma, other, or unknown. Reasons categorized as “other” are reviewed by two physicians to determine whether the admission is likely COVID-19–related.

^¶¶^ Defined as one or more of the following: chronic lung disease, chronic metabolic disease, blood disorder/hemoglobinopathy, cardiovascular disease, neurologic disorder, immunocompromising condition, renal disease, gastrointestinal/liver disease, rheumatologic/autoimmune/inflammatory condition, obesity, feeding tube dependency, and wheelchair dependency.

*** Includes children with development delay (211), seizure disorders (139), cerebral palsy (62), and other neurologic disorders such as Down Syndrome, neural tube defect, neuropathy, paralysis, and mitochondrial disorders.

^††† ^Includes children with obstructive sleep apnea (74), oxygen dependency (18), bronchopulmonary dysplasia (22), and other chronic lung conditions such as airway abnormality, tracheostomy dependency, restrictive lung disease, pulmonary fibrosis, chronic obstructive pulmonary disease, idiopathic lung disease, chronic bronchitis, bronchiolitis obliterans, and bronchiectasis.

^§§§ ^Includes children with congenital heart disease (55), aortic regurgitation (45), aortic stenosis (30) and other cardiological disorders such as cardiomyopathy and dysrhythmias.

^¶¶¶^ Includes children with sickle cell anemia (81), asplenia (20), thrombocytopenia (11), and other blood disorders such as thalassemia, coagulopathy, and myelodysplastic syndromes.

**** Includes children with immunosuppressive therapy (70), leukemia (40), immunoglobulin deficiency (13), and other immunocompromising conditions including lymphoma and solid organ malignancies.

^††††^ Includes children with thyroid dysfunction (20), adrenal disorders (13), and other metabolic conditions such as pituitary dysfunction, inborn errors of metabolism, parathyroid dysfunction, and glycogen or other storage diseases.

^§§§§^ Includes children with rheumatoid arthritis (32), lupus erythematosus (four), systemic sclerosis (four), and other autoimmune or inflammatory disorders such as Kawasaki disease and juvenile idiopathic arthritis.

^¶¶¶¶ ^Includes children with ulcerative colitis (six), Crohn’s disease (two), chronic liver disease (two), and other GI/liver diseases such as nonalcoholic fatty liver disease, hepatitis B, and esophageal strictures.

***** Includes children with renal insufficiency (13), nephrotic syndrome (five), and other renal diseases, such as glomerulonephritis, polycystic kidney disease, and end stage renal disease.

^†††††^ Excludes genetic diseases listed above.

^§§§§§^ Across periods, the number of children aged 5–11 years tested for additional viral pathogens was 654 (55%); 85 (12%) had received a positive test result. Positive test results include those for respiratory syncytial virus (13), influenza (four), rhinovirus/enterovirus (52), and other viruses (19).

^¶¶¶¶¶ ^Hospitalization outcomes are not mutually exclusive; patients can be included in more than one category.

Across periods, 32% of hospitalized children aged 5–11 years had severe COVID-19; 44% of Black children and 26% of Hispanic children experienced severe disease, compared with 22% of White children, but the association between severe COVID-19 and race or Hispanic ethnicity was not statistically significant (Table 2). The risk for severe COVID-19 among hospitalized children was significantly higher among those with diabetes (aRR = 2.5) and obesity (aRR = 1.2). Risk for severe disease was lower among children with asthma (aRR = 0.8), immunocompromising conditions (aRR = 0.7), and those hospitalized during the Delta-predominant (aRR = 0.8) and Omicron-predominant periods (aRR = 0.6). Other conditions were not significantly associated with severe COVID-19 among hospitalized children.

**TABLE 2:** Demographic characteristics, underlying conditions, and variant periods associated with severe COVID-19* among children aged 5–11 years hospitalized with COVID-19 as the primary reason for admission^†^ — COVID-NET, March 1, 2020–February 28, 2022

Characteristic	No. (%) of hospitalized children^§^				Bivariate models	Multivariable models
	Severe disease		No severe disease		RR (95% CI)	aRR (95% CI)
**Age, yrs, median (IQR)**	304	8 (6–10)^¶^	639	8 (6–10)^¶^	1.02 (1.00–1.04)	1.02 (0.99–1.05)
**Sex**						
Male	165	53.5	345	52.9	1.02 (0.86–1.21)	1.03 (0.87–1.21)
Female	139	46.5	294	47.1	Ref	Ref
**Race/Ethnicity**						
White, non-Hispanic	67	22.4	180	28.0	Ref	Ref
Black, non-Hispanic	134	43.6	224	34.9	1.36 (0.85–2.18)	1.38 (0.95–2.00)
Asian or Pacific Islander, non-Hispanic	13	4.4	28	4.6	1.15 (0.44–3.01)	1.13 (0.47–2.76)
Hispanic	78	25.9	172	27.2	1.13 (0.79–1.63)	1.15 (0.70–1.88)
Unknown/Other races**	12	3.7	35	5.2	0.91 (0.35–2.36)	0.97 (0.41–2.27)
**Underlying medical conditions^†^**						
Diabetes mellitus^††^	34	12.2	18	3.3	2.16 (1.46–3.20)	2.47 (2.12–2.87)
Chronic lung disease^§§^	45	15.2	69	10.8	1.29 (0.89–1.88)	1.35 (0.81–2.24)
Feeding tube dependence	31	10.3	35	5.9	1.46 (1.29–1.66)	1.28 (0.97–1.69)
Neurologic disorder	91	31.3	159	24.9	1.24 (1.03–1.50)	1.23 (0.92–1.63)
Chronic metabolic disease^§§^	14	4.6	22	3.5	1.22 (0.81–1.85)	1.20 (0.85–1.70)
Obesity	87	27.1	151	23.7	1.13 (1.00–1.28)	1.19 (1.06–1.34)
Cardiovascular disease	42	14.4	84	13.5	1.05 (0.91–1.21)	0.99 (0.82–1.19)
Asthma	64	21.0	177	26.7	0.80 (0.66–0.97)	0.75 (0.65–0.86)
Immunocompromising condition	18	6.1	71	11.7	0.59 (0.50–0.70)	0.68 (0.60–0.78)
Blood disorder	18	6.2	81	12.6	0.55 (0.28–1.12)	0.56 (0.29–1.07)
Other^¶¶^	39	13.3	80	12.9	1.02 (0.90–1.16)	0.91 (0.71–1.17)
**Variant periods**						
Pre-Delta	154	47.7	266	36.4	Ref	Ref
Delta-predominant	112	34.8	251	35.7	0.82 (0.72–0.93)	0.83 (0.69–0.99)
Omicron-predominant	38	17.5	122	28.0	0.59 (0.47–0.74)	0.57 (0.43–0.76)

**Abbreviations:** aRR = adjusted risk ratio; COVID-NET = COVID-19–Associated Hospitalization Surveillance Network; ICU = intensive care unit; Ref = referent group; RR = risk ratio.

* Defined as requiring ICU admission or invasive mechanical ventilation, or in-hospital death.

^†^ Among sampled patients, COVID-NET collects data on the primary reason for admission to differentiate hospitalizations of patients with laboratory-confirmed SARS-CoV-2 infection who are likely admitted primarily for COVID-19 illness rather than for other reasons. During chart review, if the surveillance officer finds that the chief complaint or history of present illness mentions fever or respiratory illness, COVID-19–like illness, or suspected COVID-19, then the case is categorized with COVID-19–related illness as the primary reason for admission. Reasons for admission that are likely primarily not related to COVID-19 include the following categories: inpatient surgery or procedures, psychiatric admission requiring acute medical care, trauma, other, or unknown. Reasons categorized as “other” are reviewed by two physicians to determine whether the admission is likely COVID-19–related.

^§^ Data are from a weighted sample of hospitalized children with completed medical record abstractions. Sample sizes presented are unweighted with weighted percentages.

^¶^ Age was modeled as a continuous variable and presented as the median and IQR.

** Includes non-Hispanic persons reported as other, multiple races, and unknown race or ethnicity.

^††^ Includes type 1 and type 2 diabetes mellitus.

^§§ ^Chronic lung disease excludes asthma and chronic metabolic disease excludes diabetes mellitus.

^¶¶^ Includes liver disease; renal disease; rheumatologic, autoimmune, and inflammatory conditions; and other conditions specified on the case report form.

## Discussion

Peak weekly COVID-19–associated hospitalization rates among children aged 5–11 years were higher during the Omicron-predominant period than during the Delta-predominant period. During Omicron predominance, shortly after the Food and Drug Administration authorized COVID-19 vaccination for this age group, population-based hospitalization rates among unvaccinated children were twice as high as were those among vaccinated children. Most hospitalized children were unvaccinated, and nearly one in three were Black. Approximately one third had no underlying medical conditions, and nearly one fifth required ICU admission. The potential for serious illness among children aged 5–11 years, including those with no underlying health conditions, highlights the importance of vaccination among this age group.

Vaccination eligibility was expanded to include children aged 5–11 years on November 2, 2021. As of March 5, 2022, 32% of children in this age group had completed a COVID-19 primary vaccination series.^ ¶¶¶¶^ In this study, approximately one half (53%) of unvaccinated hospitalized children were Black or Hispanic, two groups known to have lower vaccination rates (*3*). Implementing strategies that result in equitable receipt of COVID-19 vaccine among children is a public health priority.

The finding that hospitalization rates in unvaccinated children were double those of vaccinated children suggests that vaccines are effective in preventing COVID-19–associated morbidities. This is consistent with recent studies, which suggest that vaccination reduces the risk for Omicron infection, protects against COVID-19–associated illness among children aged 5–11 years and prevents multisystem inflammatory syndrome in children, a severe postinfectious hyperinflammatory condition with a higher incidence in this age group than in other age groups (*4*–*7*).

Consistent with other studies, this analysis demonstrated that the Omicron-predominant period was associated with less severe disease among hospitalized children (*8*). However, both population-based peak hospitalization and ICU admission rates were higher during the Omicron-predominant period compared with those during the Delta-predominant period, likely because of the high transmissibility of the Omicron variant and greater number of persons infected. Although a higher proportion of children hospitalized with laboratory-confirmed SARS-CoV-2 infection were admitted for reasons that were not likely primarily COVID-19–related during the Omicron period compared with the Delta period, most children admitted during both periods were hospitalized primarily for COVID-19. These findings suggest that incidental admissions do not account for the increase in hospitalization rates observed during the Omicron period and reinforce that children continued to experience serious COVID-19 illness.

As in previous investigations, diabetes and obesity were associated with increased risk for severe COVID-19 in children (*2*). One third of hospitalized children aged 5–11 years had underlying neurologic disorders during the Omicron-predominant period, an increase from previous periods. Neurologic disorders have been shown to increase risk for severe illness in other respiratory diseases such as influenza (*9*). Consistent with findings from influenza-associated hospitalizations, this study found that some underlying medical conditions, including asthma and immunocompromising conditions, were not associated with increased risk for severe COVID-19, which might be explained by a lower threshold for hospital admission in children with these conditions (*10*).

The findings in this report are subject to at least five limitations. First, COVID-19–associated hospitalizations might have been missed because of testing practices and availability. Second, stratification of hospitalization rate by vaccination status is subject to error if misclassification of vaccination status occurred. Third, analyses based on vaccination status are biased toward the null because partially vaccinated children were grouped with unvaccinated children. Fourth, primary reason for admission was not always clear, and medical charts might not completely capture underlying conditions, potentially resulting in misclassification. Finally, COVID-NET catchment areas include approximately 10% of the U.S. population; thus, these findings might not be generalizable to the rest of the United States.

Potential for serious disease requiring hospitalization, ICU admission, or IMV among children aged 5–11 years reinforces the importance of increasing vaccination coverage among this population. Black children accounted for the highest percentage of unvaccinated children in this analysis and represented one third of COVID-19–associated hospitalizations in this age group. Increasing COVID-19 vaccination coverage among children aged 5–11 years, with particular attention to racial and ethnic minority groups disproportionately affected by COVID-19, is critical to reducing COVID-19–associated morbidity.*****

**Summary**

**What is already known about this topic?**

COVID-19 can cause severe illness in children. Children aged 5–11 years became eligible for COVID-19 vaccination on November 2, 2021.

**What is added by this report?**

During the period of Omicron predominance (December 19, 2021–February 28, 2022), COVID-19–associated hospitalization rates in children aged 5–11 years were approximately twice as high among unvaccinated as among vaccinated children. Non-Hispanic Black children represented the largest group of unvaccinated children. Thirty percent of hospitalized children had no underlying medical conditions, and 19% were admitted to an intensive care unit. Children with diabetes and obesity were more likely to experience severe COVID-19.

**What are the implications for public health practice?**

Increasing COVID-19 vaccination coverage among children aged 5–11 years, particularly among racial and ethnic minority groups disproportionately affected by COVID-19, can prevent COVID-19–associated hospitalization and severe outcomes.

## Footnotes

* https://www.fda.gov/news-events/press-announcements/fda-authorizes-pfizer-biontech-covid-19-vaccine-emergency-use-children-5-through-11-years-age; https://www.cdc.gov/media/releases/2021/s1102-PediatricCOVID-19Vaccine.html

† Omicron became the predominant variant during the week ending December 25, 2021 at 74% of sequenced isolates. https://covid.cdc.gov/covid-data-tracker/#variant-proportions

§
https://www.cdc.gov/coronavirus/2019-ncov/covid-data/covid-net/purpose-methods.html

¶ Vaccinated children aged 5–11 years were defined as those who had received the final dose in their primary series ≥14 days before receiving a positive SARS-CoV-2 test result associated with their hospitalization. Children who had received only 1 vaccine dose ≥14 days before the SARS-CoV-2 test date or had received a single dose of vaccine <14 days before the positive SARS-CoV-2 test results were considered partially vaccinated; these children were not included in rates and were grouped with unvaccinated children in other analyses.

**
https://covid.cdc.gov/covid-data-tracker/#covidnet-hospitalizations-vaccination

†† California, Colorado, Connecticut, Georgia, Iowa, Maryland, Michigan, Minnesota, New Mexico, New York, Ohio, Oregon, Tennessee, and Utah.

§§ Rates are calculated using the National Center for Health Statistics’ vintage 2020 bridged-race postcensal population estimates for the counties included in surveillance. https://www.cdc.gov/nchs/nvss/bridged_race.htm

¶¶ COVID-NET sites, through agreements with state health departments and other partners, collect COVID-19 vaccination information on COVID-19–associated hospitalizations through state-based vaccination registries. When possible, sites collect COVID-19 vaccination status on all persons with COVID-19 cases who are hospitalized, including the number of vaccine doses received, the vaccine product, and dates of vaccine administration. Vaccination information was not available for Iowa, Maryland, and Michigan and only available for sampled cases in Minnesota.

*** During December 2021–February 2022, sites sampled pediatric patients at rates of 12%–100%. Random numbers (1–100) were automatically generated and assigned to each patient on entry into the surveillance database to produce random samples of hospitalized patients for medical record abstraction. Percentages were weighted to account for the probability of selection for sampled patients.

††† Among sampled cases, COVID-NET collects data on the primary reason for admission to differentiate hospitalizations of patients with laboratory-confirmed SARS-CoV-2 infection who are likely admitted primarily for COVID-19 illness from those admitted for other reasons, including inpatient surgery or trauma. During chart review, if the surveillance officer finds that the chief complaint or history of present illness mentions fever/respiratory illness, COVID-19–like illness, or a suspicion for COVID-19, then the case is categorized with COVID-19–related illness as the primary reason for admission.

§§§ COVID-19–related symptoms included respiratory symptoms (congestion/runny nose, cough, hemoptysis/bloody sputum, shortness of breath/respiratory distress, sore throat, upper respiratory infection, influenza-like illness, and wheezing) and nonrespiratory symptoms (abdominal pain, altered mental status/confusion, anosmia/decreased smell, chest pain, conjunctivitis, diarrhea, dysgeusia/decreased taste, fatigue, fever/chills, headache, muscle aches/myalgias, nausea/vomiting, rash, and seizures). Symptoms were abstracted from the medical chart and might be incomplete.

¶¶¶ Thirteen underlying conditions were considered, including airway abnormality, asthma, blood disorders, cardiovascular disease, developmental delay, diabetes mellitus (type 1 or 2), feeding tube dependence, immunocompromising conditions, obesity (body mass index [kg/m^2^] ≥95th percentile for age and sex based on CDC growth charts; *International Classification of Diseases*, *Tenth Revision*, *Clinical Modification* codes for obesity; or obesity selected on the case report form), nonasthma chronic lung disease, nondiabetes chronic metabolic disease, nondevelopmental delay neurologic disorders, or other conditions (gastrointestinal or liver disease; renal disease; or rheumatologic, autoimmune, or inflammatory disease).

*****
https://www.cdc.gov/coronavirus/2019-ncov/vaccines/stay-up-to-date.html
